# Characterization of Pancreatic Cancer Tissue Using Multiphoton Excitation Fluorescence and Polarization-Sensitive Harmonic Generation Microscopy

**DOI:** 10.3389/fonc.2019.00272

**Published:** 2019-04-17

**Authors:** Danielle Tokarz, Richard Cisek, Ariana Joseph, Ahmad Golaraei, Kamdin Mirsanaye, Serguei Krouglov, Sylvia L. Asa, Brian C. Wilson, Virginijus Barzda

**Affiliations:** ^1^Department of Chemistry, Saint Mary's University, Halifax, NS, Canada; ^2^Department of Physics, University of Toronto, Toronto, ON, Canada; ^3^Department of Chemical and Physical Sciences, University of Toronto Mississauga, Mississauga, ON, Canada; ^4^Princess Margaret Cancer Centre, University of Toronto, Toronto, ON, Canada; ^5^University Health Network, University of Toronto, Toronto, ON, Canada; ^6^Department of Medical Biophysics, University of Toronto, Toronto, ON, Canada

**Keywords:** collagen, optical pathology, medical imaging, non-linear optical polarimetry, non-linear optical microscopy

## Abstract

Thin tissue sections of normal and tumorous pancreatic tissues stained with hematoxylin and eosin were investigated using multiphoton excitation fluorescence (MPF), second harmonic generation (SHG), and third harmonic generation (THG) microscopies. The cytoplasm, connective tissue, collagen and extracellular structures are visualized with MPF due to the eosin stain, whereas collagen is imaged with endogenous SHG contrast that does not require staining. Cellular structures, including membranous interfaces and nuclear components, are seen with THG due to the aggregation of hematoxylin dye. Changes in the collagen ultrastructure in pancreatic cancer were investigated by a polarization-sensitive SHG microscopy technique, polarization-in, polarization-out (PIPO) SHG. This involves measuring the orientation of the linear polarization of the SHG signal as a function of the linear polarization orientation of the incident laser radiation. From the PIPO SHG data, the second-order non-linear optical susceptibility ratio, χ^(2)^_*zzz*_'/χ^(2)^_*zxx*_', was obtained that serves as a structural parameter for characterizing the tissue. Furthermore, by assuming C_6_ symmetry, an additional second-order non-linear optical susceptibility ratio, χ^(2)^_*xyz*_'/χ^(2)^_*zxx*_', was obtained, which is a measure of the chirality of the collagen fibers. Statistically-significant differences in the χ^(2)^_*zzz*_'/χ^(2)^_*zxx*_' values were found between tumor and normal pancreatic tissues in periductal, lobular, and parenchymal regions, whereas statistically-significant differences in the full width at half maximum (FWHM) of χ^(2)^_*xyz*_'/χ^(2)^_*zxx*_' occurrence histograms were found between tumor and normal pancreatic tissues in periductal and parenchymal regions. Additionally, the PIPO SHG data were used to determine the degree of linear polarization (DOLP) of the SHG signal, which indicates the relative linear depolarization of the signal. Statistically-significant differences in DOLP values were found between tumor and normal pancreatic tissues in periductal and parenchymal regions. Hence, the differences observed in the χ^(2)^_*zzz*_'/χ^(2)^_*zxx*_' values, the FWHM of χ^(2)^_*xyz*_'/χ^(2)^_*zxx*_' values and the DOLP values could potentially be used to aid pathologists in diagnosing pancreatic cancer.

## Introduction

Pancreatic cancer is currently the third-leading cause of death from cancer in the USA (1). It is rarely detected at an early stage, since symptoms are often not present until the cancer has spread to other organs, thereby making it one of the deadliest cancers. A diagnosis of cancer is confirmed by visualizing a stained thin section of tissue obtained at biopsy or surgical resection using bright-field microscopy. The initiation of cancer has been attributed to the progressive accumulation of somatic mutations in epithelial cells (2). In addition, recent studies also implicate the tumor microenvironment, including the extracellular matrix (ECM), blood vasculature and inflammatory cells and fibroblasts, in cancer promotion (3–7). During cancer initiation and progression, mechanisms responsible for ensuring normal organ development and function are deregulated, leading to ECM disorganization (8), so that a technique that identifies and quantifies structural details in the ECM as well as permits the distinction of cells present in the ECM would improve cancer diagnosis.

Collagen, the most abundant protein in the ECM, gives rise to strong second harmonic generation (SHG) signals without staining (9–14), so that the collagen distribution within the pancreatic tissue microenvironment can be visualized with SHG microscopy (15–17). Cells present in the ECM, and stained with standard histological dyes such as hematoxylin and eosin (H&E), give rise to strong multiphoton excitation fluorescence (MPF) and third harmonic generation (THG) signals (18, 19). Therefore, quantification of nuclei shape and size can be performed with THG microscopy (19, 20). Additionally, polarization-sensitive SHG microscopy can be performed to extract collagen structural information (21–30). Previously, polarization-sensitive SHG microscopy has been used to determine structural differences in normal and malignant breast tissue (31–33), models of ovarian cancer (34–37), colon cancer (38), non-small cell lung carcinoma (39), squamous-cell carcinoma (40), and follicular variant papillary thyroid carcinoma, as well as classical papillary thyroid carcinoma (41, 42). Of those studies, malignant breast tissue (32), non-small cell lung carcinoma (39), and thyroid carcinoma (41, 42) were performed using polarization-in, polarization-out (PIPO) SHG microscopy. Currently, faster polarization-sensitive SHG microscopy techniques are being developed with the use of liquid-crystal (43) and electro-optic modulators (44, 45) or through the use of multiple detectors to deduce Stokes parameters (46–48). These techniques are promising for dynamic studies.

In this paper, SHG, MPF, THG, and polarization-sensitive SHG microscopy are used to visualize normal and tumorous pancreatic tissue. The SHG intensities and polarization parameters related to the structure of collagen are extracted including the χ^(2)^_*zzz*_'/χ^(2)^_*zxx*_' value associated with the distribution of fibers, as well as the χ^(2)^_*xyz*_'/χ^(2)^_*zxx*_' value related to the chiral structure of collagen. Additionally, the degree of linear polarization (DOLP) of SHG associated with the presence of disorder of collagen fibers is determined. The THG intensities of cells present in normal and tumorous pancreatic tissues are also measured. The study investigates the possibility of whether pancreatic cancer diagnosis by identifying cancer cells using THG imaging or polarization-resolved SHG imaging of the extracellular matrix is viable. For this, the SHG and THG intensities as well as the SHG polarization parameters in tumor tissue were compared with those in three distinct regions in normal pancreatic tissue all commonly found in a single tissue section. The architecture of normal pancreatic tissue consists of ducts, fibrous septa that delineate lobes and lobules, and parenchyma composed of acinar cells and scattered endocrine cells; where each tissue type has a distinct biological function. Due to the varying architecture of these units, it is important to compare any differences in the SHG and THG intensities, and the SHG polarization parameters amongst the normal tissue to understand their variability across normal tissue elements before comparing them to those measured from tumor taken from the same area of the pancreas. In this study, all tumors were adenocarcinomas that originate from ducts or ductules but infiltrate to involve all components of the pancreas. Other tumors that originate in pancreas may have different properties and a future study of *in situ* tumor is likely needed to determine whether the tumor tissue retains a structural remnant of its originating tissue.

## Methods and Materials

### Histology Sample Preparation

Samples of normal human pancreas from five patients and pancreatic ductal adenocarcinoma tissue samples from ten patients were obtained with informed consent and institutional approval (University Health Network Toronto, Canada). The tissues were handled as per standard clinical histology protocols. Thin sections (5 μm) were cut from formalin-fixed paraffin-embedded tissues, mounted on glass slides and stained with H&E for histopathologic analysis. All slides were scanned at ×20 magnification using a whole-slide scanner (ScanScope XT: Leica Biosystems, Germany).

In each slide, 110 × 110 μm regions of interest, as identified by a pathologist (S.L.A), were scanned. Identification was performed by assessing the tissue architecture and cytology of the tumor cells in the high resolution bright-field microscopy images. A total of 47 tumor and 51 normal regions were imaged to determine quantitative differences between tumor and normal tissue. Two-tailed *t*-tests of statistical significance were performed.

### Non-linear Optical Microscope Setup

A custom-built Yb:KGW laser operating at 1028 nm, 14.3 MHz repetition rate and ~450 fs pulse duration (49) was used for MPF, SHG and THG imaging. This was coupled to a custom-built non-linear optical microscope, described previously (50, 51). Briefly, galvanometric scanning mirrors (VM1000, Cambridge Technology, USA) were used to raster scan the beam through a 0.75 numerical aperture (NA) air objective lens (Plan-Apochromat 20×, Carl Zeiss AG, Germany) that does not alter polarization at up to 10 frames per second. MPF, SHG and THG signals were collected in transmission mode through a custom-built 0.85 NA collection objective lens (Omex Technologies, USA). A pulse energy of ~0.05 nJ was used for MPF imaging and a pulse energy of ~1 nJ was used for SHG and THG imaging.

For the MPF channel, the 525–630 nm band was collected (FF01-578/105 filter, Semrock Inc., USA), which included mostly eosin fluorescence. SHG was filtered with an interference filter centered at 514.5 nm with 10 nm bandwidth (F10-514.5, CVI Laser Optics, USA). For both MPF and SHG imaging, the intense excitation light requires additional filtering using a Schott color glass filter (BG39, CVI Laser Optics, USA). THG signals were selected by a single bandpass interference filter of 340 nm with 10 nm bandwidth (F10-340, CVI Laser Optics, USA). The signals were measured using single-photon-counting detectors (H7421-40, Hamamatsu Photonics K.K., Japan).

For polarization measurements of SHG signals, the polarization-in, polarization-out (PIPO) technique was used, as previously described (52). Briefly, the microscope was modified by addition of a polarization-state generator (PSG), consisting of a linear polarizer (IR 1100 BC4, Laser Components, Germany) followed by a half-wave plate (532GR-42, Comar Optics Ltd., United Kingdom) placed immediately before the excitation objective lens for rotation of the incident laser polarization. To measure the polarization of the SHG signal, a polarization-state analyzer (PSA) was used, consisting of a linear polarizer (10LP-VIS-B, Newport Corporation, USA) located after the collection objective lens. A typical PIPO SHG measurement consisted of recording an SHG image at 9 emission polarization angles for each of 9 half-wave plate angles. Every 9 images an additional image was obtained at reference polarizer and analyzer angles as a control. A pulse energy of ~0.5 nJ was used for PIPO SHG imaging. To measure the variation in SHG intensity between normal and tumor tissues, the PSG was modified by adding a quarter-wave plate (WPQ05M-1064, Thorlabs, Inc., USA) just before the excitation objective lens in order to obtain circularly polarized light.

### Second-Order Non-linear Optical Susceptibility Ratios and the Degree of Linear Polarization From Polarization-Sensitive SHG Measurements

The second-order non-linear optical susceptibility tensor components ratio of collagen fibers was determined from PIPO SHG measurements as described previously (52, 53). Briefly, a laboratory Cartesian coordinate system (*XYZ*) was defined with respect to the principal propagation direction of the laser (*Y*), where *XZ* is defined as the image plane. The average orientation of collagen fibers in a voxel was defined by modified spherical angles, where δ is the average in-plane fiber orientation measured from the *Z*-axis, and α is the out-of-plane tilt angle of the fiber.

Assuming C_6_ symmetry, the SHG intensity can be described as a function of the laser electric field polarization orientation (θ) and the orientation of the analyzer (ϕ) (54):

(1) $$I_{2\omega }\propto {\left|\begin{array}{l} \frac{\chi_{xxz^{\prime }}^{\left(2\right)}}{\chi_{zxx^{\prime }}^{\left(2\right)}}sin\left(\varphi -\delta \right)sin2\left(\theta -\delta \right)+cos\left(\varphi -\delta \right)sin^{2}\left(\theta -\delta \right)+\frac{\chi_{zzz^{\prime }}^{\left(2\right)}}{\chi_{zxx^{\prime }}^{\left(2\right)}}cos\left(\varphi -\delta \right)cos^{2}\left(\theta -\delta \right)\\ \;+2\frac{\chi_{xyz^{\prime }}^{\left(2\right)}}{\chi_{zxx^{\prime }}^{\left(2\right)}}cos\left(\varphi -\delta \right)sin\left(\theta -\delta \right) \end{array}\right|}^{2}$$

The primed coordinate denotes the molecular susceptibility projected onto the image plane. Equation (1) shows that 3 second-order non-linear optical susceptibility tensor component ratios of an arbitrarily oriented fiber could be deduced from PIPO SHG measurements. It was assumed that χ^(2)^_*xxz*_'*/*χ^(2)^_*zxx*_' = 1 in order to reduce the free-parameter space for more accurate fitting, and is valid as long as collagen behaves as rod-like structures with a dominant χ^(2)^_*zzz*_ (55). The molecular second-order non-linear optical susceptibility tensor component ratios (χ^(2)^_*zzz*_*/*χ^(2)^_*zxx*_ and χ^(2)^_*xyz*_*/*χ^(2)^_*zxx*_) of an arbitrarily oriented fiber at an angle α from the imaging plane is related to the measured susceptibility ratios (*R* or χ^(2)^_*zzz*_'*/*χ^(2)^_*zxx*_' and *C* or χ^(2)^_*xyz*_'*/*χ^(2)^_*zxx*_', respectively) as:

(2) $$\begin{array}{l} R=\frac{\chi_{zzz^{\prime }}^{\left(2\right)}}{\chi_{zxx^{\prime }}^{\left(2\right)}}=\frac{\chi_{zzz}^{\left(2\right)}}{\chi_{zxx}^{\left(2\right)}}{cos}^{2}\alpha +3{sin}^{2}\alpha \\ \;C=\frac{\chi_{xyz^{\prime }}^{\left(2\right)}}{\chi_{zxx^{\prime }}^{\left(2\right)}}=\frac{\chi_{xyz}^{\left(2\right)}}{\chi_{zxx}^{\left(2\right)}}sin\alpha \end{array}$$

The equations show that at low **α** angles, *R* is representative of its molecular counterpart, while the measured *C* parameter would be near 0, conversely larger measured *C* values, closer to their molecular counterparts are only expected from fibers at higher **α**. Alternatively, when non-chiral cylindrical (C_6v_) symmetry is assumed, χ^(2)^_*xyz*_'*/*χ^(2)^_*zxx*_' = 0 in Equation (1) (52, 53). In addition to extracting the *R* and *C* values, a third parameter known as the degree of linear polarization (DOLP) was extracted from:

(3) $$\begin{array}{r} \begin{array}{c} DOLP=\frac{\sqrt{{s_{1}^{2}+s}_{2}^{2}}}{s_{0}} \end{array} \end{array}$$

Stokes parameters, *s*_0_, *s*_1_ and *s*_2_, describe the SHG polarization, defined as: *s*_0_ = *I*_0_+*I*_90_, *s*_1_ = *I*_0_−*I*_90_, and *s*_2_ = *I*_45_−*I*_−45_, where *I*_*a*_ is the SHG intensity at the analyzer angle *a*. According to double-Stokes-Mueller polarimetry theory (56), a measurement of the DOLP depends on the angle between the incident polarization of the laser and the measured collagen fiber angle (δ). When the laser polarization orientation is 90° from the collagen axis, the SHG signal intensity is reduced and noise dominates the signal, causing the DOLP to be unreliable. However, meaningful data were obtained by averaging together DOLP measurements at 8 incident laser polarizations (0, 22.5, 45, 67.5, 90, 112.5, 135, and 157.5°), and for each one, two DOLP calculations were averaged, one using measurements at analyzer angles 0, 45, 90, and 135° and another at 22.5, 67.5, 112.5, and 157.5°. Analysis and fitting of PIPO SHG data with Equations (1)–(3) were performed using custom MATLAB (The Mathworks, Inc.) software. Since the tissues were thin (5 μm), birefringence was neglected (53).

## Results

### Non-linear Optical Imaging of Normal and Tumor Pancreatic Tissues

**Figure 1:**
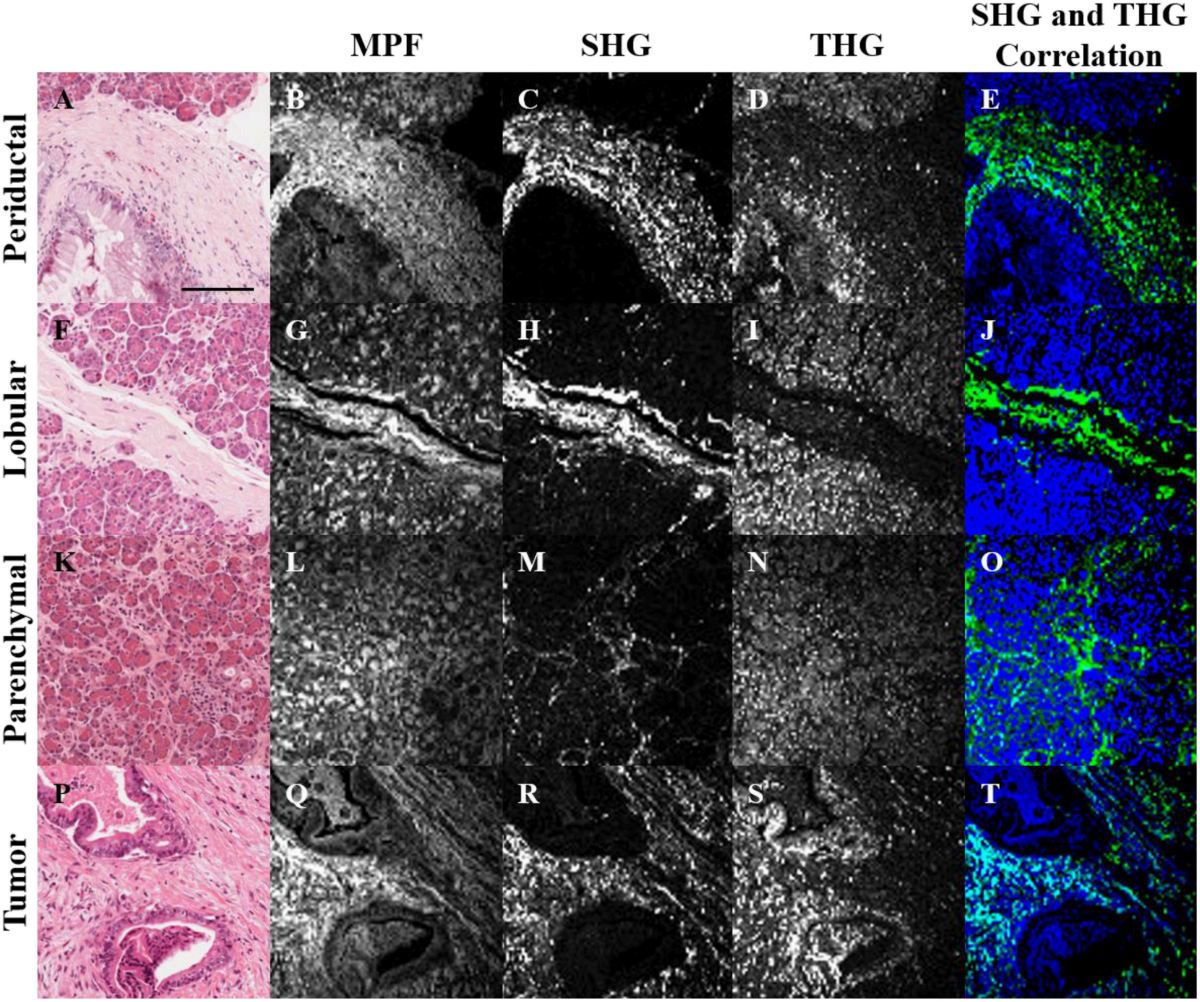
Bright-field **(A,F,K,P)**, MPF **(B,G,L,Q)**, SHG **(C,H,M,R)**, and THG **(D,I,N,S)** images of periductal **(A–E)**, lobular **(F–J)** and parenchymal **(K–O)** regions of normal pancreas, as well as tumor tissue **(P–T)**. SHG and THG images were correlated **(E,J,O,T)**, where regions with correlation between SHG and THG are visualized in cyan, while uncorrelated SHG and THG are visualized in green and blue, respectively. The scale bar (in **A**) represents 125 μm.

H&E stained histopathology sections of normal and tumorous human pancreatic tissue were imaged with MPF, SHG and THG microscopy (Figure 1). In general, the MPF signal from H&E stained tissues visualizes the entire extracellular matrix due to the eosin staining (Figures 1B,G,L,Q). The SHG signal is generated intrinsically from collagen (Figures 1C,H,M,R), while the THG signal is predominantly generated by hemalum complexes from chromatin-bound hematoxylin (19). Hence, THG visualizes nuclear material, including nucleoli and the nuclear envelope.

Structural cross-correlation image analysis between the 3 imaging modalities was performed using an overlap and threshold algorithm (57, 58). In normal tissue, the SHG and THG signals were largely uncorrelated (Figures 1E,J,O), but more correlation was observed in tumor (Figure 1T), likely due to the increased content of chromatin in the nuclei of collagenous tumor regions. Further rigorous analysis is needed in order to quantify and determine the statistical significance of this observation. Analysis of the correlation between MPF and the other signals was not useful, since the MPF from eosin is not selective for collagen or nuclei (Figures 1D,I,N,S).

### SHG Intensity From Normal and Cancerous Pancreatic Tissues

**Figure 2:**
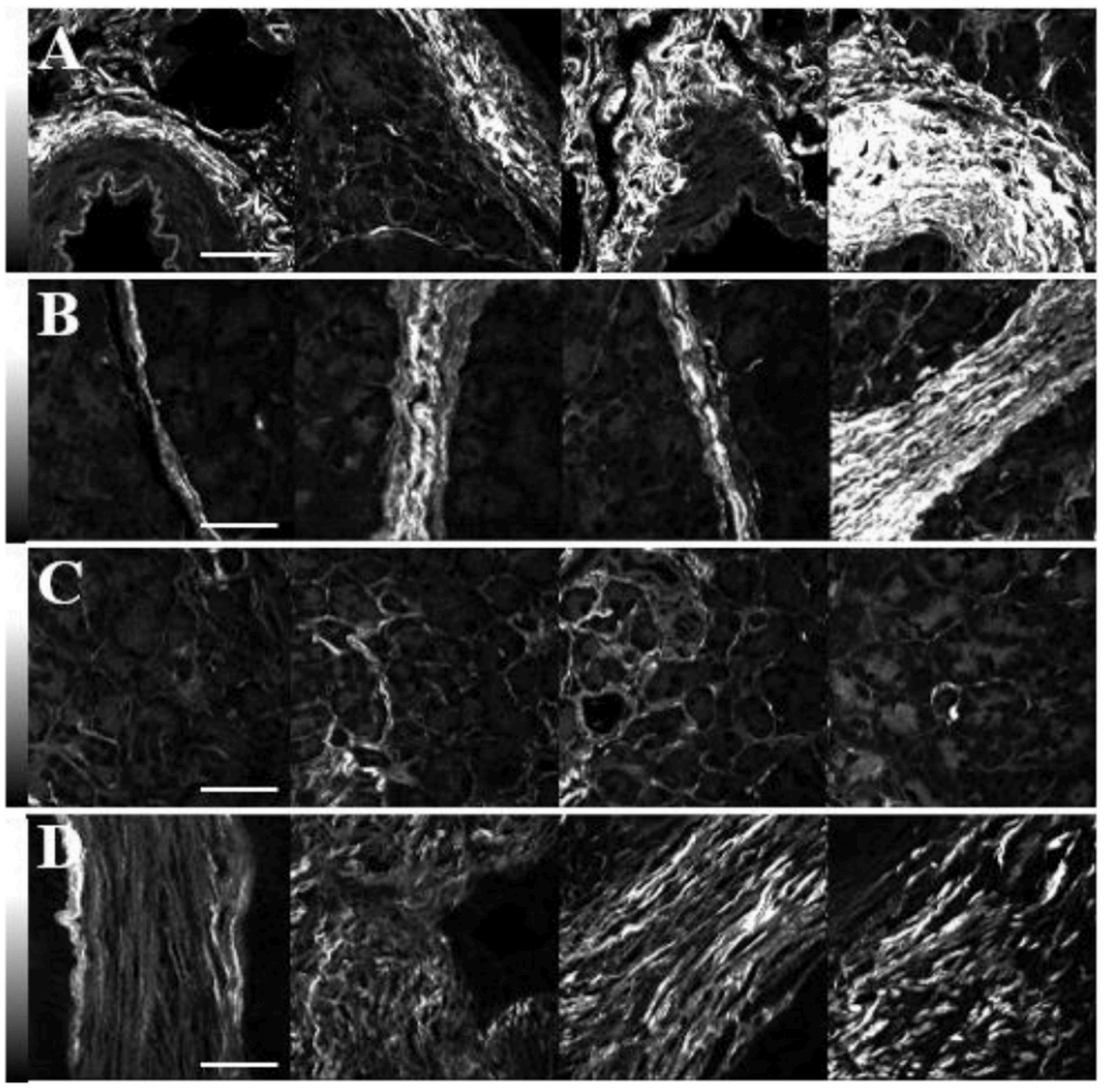
SHG intensity comparison of collagen in the periductal **(A)**, lobular **(B)** and parenchymal **(C)** regions of normal pancreas as well as in tumor **(D)**. The imaging laser was circularly polarized and identical conditions were used for all the images. The scale bars represent 50 μm, and the intensity scale bars represent the SHG signal in each pixel.

Representative images of the SHG intensity of collagen in the normal periductal, lobular, and parenchymal tissues and in tumor are shown in Figures 2A–D. The values are independent of the fiber orientation angle, δ, since circularly polarized laser light was used. Furthermore, in order to perform valid intensity comparisons, identical laser powers and pixel dwell times were used. In general, the SHG intensity of periductal collagen (3,250 ± 1,750 photon counts: Figure 2A) was the highest, followed by lobular (2,250 ± 1,500: Figure 2B) and parenchymal (900 ± 700: Figure 2C) tissue. The higher SHG intensity of periductal and lobular collagen is attributed to the thick and densely arranged collagen around ducts and lobes as compared to the parenchymal region where the collagen fibers are thinner and sparsely arranged (59), although collagen disorder in the focal volume may also play a role. The SHG intensity of collagen in tumor (1,700 ± 800: Figure 2D) was less than in periductal and lobular regions and typically higher than parenchymal collagen however, a statistically-significant difference was not observed.

Although variations of the highest SHG intensities are easily visualized in Figure 2, in each tissue type, the SHG intensity is highly variable. For example, the row of images in Figure 2B shows intense SHG intensity from the thick lobular collagenous region while parenchymal collagen, next to the lobular collagen, has significantly lower intensities, complicating diagnosis by using SHG intensity. Hence, measurements of the ultrastructure of collagen in normal and cancerous human pancreas tissue were performed via polarization-sensitive SHG measurements.

### Polarization-Resolved SHG Measurements Analyzed With C_6v_ Symmetry

**Figure 3:**
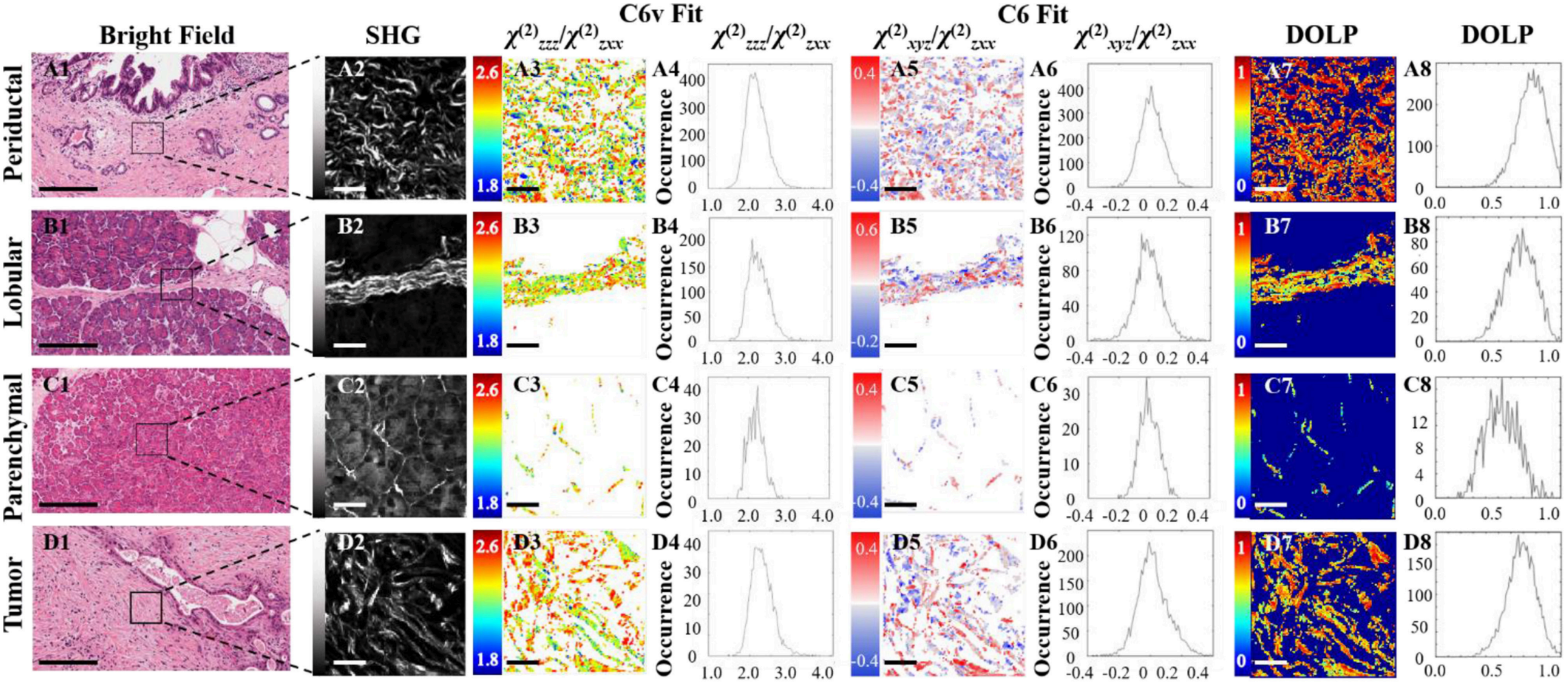
PIPO SHG of collagen in normal periductal **(A)**, lobular **(B)** and parenchymal **(C)** pancreas tissues, and in pancreatic cancer **(D)**. Bright-field images **(A1,B1,C1,D1)** where the scale bar represents 200 μm, and the corresponding SHG intensity images **(A2,B2,C2,D2)** of the regions of interest are shown, together with color-coded maps of the fitted *R* values **(A3,B3,C3,D3)** assuming C_6v_ symmetry and the occurrence histograms of the *R* values **(A4,B4,C4,D4)** where the scale bar represents 25 μm. Color-coded maps of the fitted *C* values **(A5, B5, C5, D5)** and their occurrence histograms **(A6,B6,C6,D6)** assuming C_6_ symmetry are also shown, along with maps of the extracted DOLP values **(A7,B7,C7,D7)** and occurrence histograms **(A8,B8,C8,D8)** where the scale bar represents 25 μm.

Figure 3 shows typical results of PIPO SHG imaging of periductal, lobular, parenchymal, and tumor tissues. These were analyzed using C_6v_ symmetry, revealing for each pixel the structural parameter, *R*, which depends on several factors including: the ultrastructure of individual collagen fibers, the collagen disorder within the laser beam focal volume and the tilt angle, α, between the image plane and the collagen fibers Equation (2). Previously, *R* has been investigated in collagen of different animal species, including in human normal and tumor tissues to quantify the levels of collagen disorder (32, 39, 42). The *R* value ranges from ~1.1 observed from well-ordered collagen in tibia (53) to a theoretical maximum of 3 for the most disordered structures (with the assumption that χ^(2)^_*xxz*_'*/*χ^(2)^_*zxx*_' = 1). The maps of the fitted *R* values for typical normal and tumor pancreas tissue samples are seen in Figure 3 column 3, while the adjacent graphs in column 4 indicate the corresponding *R*-values occurrence histograms from which the mean *R* values as well as the width of the distribution can be calculated, as summarized in Table 1. The mean *R* values in normal periductal, lobular, and parenchymal pancreas were significantly smaller than in tumor (*p* < 0.05). The histogram widths were generally larger for normal tissue than tumor tissue, where those of periductal and lobular regions were significantly larger than those of tumor tissue (*p* < 0.02), indicating that the tumor tissue has a narrower collagen orientation distribution.

**Table 1:** *R, C*, and corresponding DOLP values (mean ± standard deviation) assuming C_6v_ symmetry and C_6_ symmetry.

**Tissue**	**Samples (Areas)**	***R* (C_**6v**_) (FWHM)**	***R* (C_**6**_)**	***C* (FWHM)**	**DOLP**
Normal periductal	5 (17)	2.11 ± 0.11^*^ (0.73 ± 0.07)^*^	2.11 ± 0.10^*^	0.01 ± 0.02 (0.16 ± 0.05)	0.76 ± 0.07^*^
Normal lobular	5 (21)	2.14 ± 0.04^*^ (0.70 ± 0.05)^*^	2.15 ± 0.04^*^	0.01 ± 0.05 (0.20 ± 0.04)	0.66 ± 0.05
Normal parenchymal	3 (13)	2.13 ± 0.03^*^ (0.66 ± 0.04)	2.12 ± 0.03^*^	0.00 ± 0.01 (0.15 ± 0.02)^*^	0.49 ± 0.02^*^
Tumor	9 (47)	2.22 ± 0.04 (0.63 ± 0.04)	2.22 ± 0.05	−0.02 ± 0.03 (0.20 ± 0.05)	0.59 ± 0.08

Values in brackets beside “Samples” represent the number of areas analyzed. Values in brackets beside the R and C headings represent the full width at half maximum (FWHM) of the occurrence histograms. The

* *indicates statistically significant differences from the corresponding tumor values (p < 0.05)*.

### Polarization-Resolved SHG Measurements Analyzed With C_6_ Symmetry

The polarization-sensitive SHG measurements were also analyzed using the more general C_6_ symmetry, in order to evaluate whether the resultant additional chiral fitting component, *C*, would be informative in the characterization of pancreatic collagen. Fitting using Equation (1) also reveals the *R* parameter from the C_6_ fit, which corresponds well with the *R* parameter from the C_6v_ fit (see Table 1). The fitted *C* values seen in Figure 3 column 5 visualize the distribution of the collagen polarity, meaning that the two colors indicate if the average tilt of fibers in the focal volume is above or below the imaging plane. These images show that collagen fibers are clustered into small positive and negative polarity regions similarly in normal and tumor samples.

The occurrence histograms of *C* values using C_6_ symmetry are shown in Figure 3 column 6. The width of the occurrence histograms is related to the distribution of collagen tilt angles. The histograms were fit with a Gaussian function and the average *C* values and the occurrence widths were obtained. The average values for normal tissues were not significantly different than that for tumor tissue, which is unsurprising as this parameter depends on the angle at which the tissue was sectioned, which was arbitrary from sample to sample. However, the widths of the *C* value occurrence histograms for normal parenchymal pancreas was significantly smaller than that of tumor tissue (*p* < 0.05), while those for normal periductal and parenchymal tissues were significantly different from normal lobular pancreas tissue when the number of regions is taken into account (*p* < 0.02 and *p* < 0.001, respectively, see Table 1), likely related to structural variations in the collagen.

Disordered collagen fibers and the presence of small collagen fiber segments within the focal volume may produce depolarized SHG signals, which can be characterized by calculating the DOLP for each pixel of the PIPO SHG images. The influence of birefringence and scattering due to SHG propagation through birefringent tissue regions can be considered as negligible, since the tissue sections are sufficiently thin (5 μm) that there is minimal cumulative retardation of the light. An additional assumption that the scattering of the polarized SHG light is negligible is also reasonable, considering that the tissue samples are all the same thickness (60). The DOLP for normal periductal and parenchymal tissues were significantly different than for tumor (*p* < 0.002 and *p* < 0.01, respectively). The values for periductal tissues were higher than for tumor, while the values for parenchymal tissues were lower than for tumor. Lower average DOLP values in tumor tissues have previously been found in classical papillary thyroid carcinoma and follicular variant of papillary thyroid carcinoma tissues (42).

### THG Signals in Nuclei of Normal and Cancerous Pancreatic Tissues

**Figure 4:**
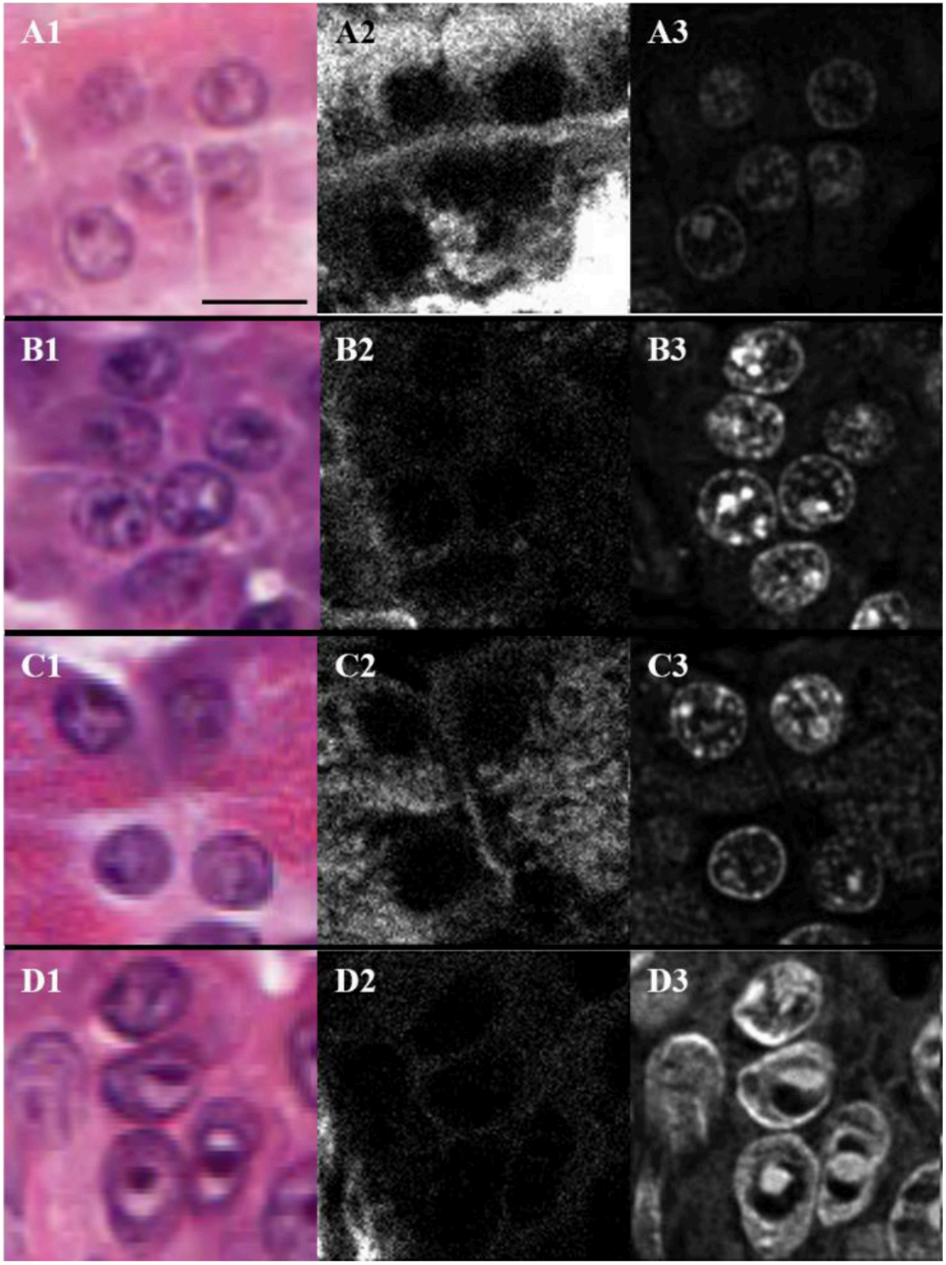
Bright-field **(A1,B1,C1,D1)**, SHG **(A2,B2,C2,D2)**, and THG **(A3,B3,C3,D3)** images of nuclei present in the periductal **(A)**, lobular **(B)** and parenchymal **(C)** regions of normal pancreas as well as in tumor **(D)** where the scale bar represents 10 μm.

The H&E stained sections were also imaged with THG using linearly polarized laser light. The analysis is valid since the THG intensity images from H&E stain were found to be independent of the orientation of laser polarization (19). Figure 4 shows typical H&E-stained bright-field (Figures 4A1,B1,C1,D1) and THG (Figures 4A3,B3,C3,D3) images and the corresponding SHG images (Figures 4A2,B2,C2,D2) for periductal (A), lobular (B), parenchymal (C) and cancerous (D) tissue regions. The nuclear envelope and nucleoli can be seen in the THG images (Figures 4A3,B3,C3,D3), due to THG enhancement by hematoxylin aggregation (19). Hence, the regions around nuclei in the different tissue types were analyzed using SHG and THG contrast. SHG from collagen lying in between nuclei is seen in Figures 4A2,B2,C2,D2 and appears strongest for periductal tissue (A), indicating higher collagen density in the matrix around nuclei. Parenchymal tissue showed lower SHG intensity, indicating reduced collagen density, while tissue surrounding nuclei in lobular and tumor tissues had the lowest collagen density. The nuclei within normal periductal, lobular and parenchymal pancreas tissues appear round and small (Figures 4A3,B3,C3), while those within tumor cells appear elongated and larger (Figure 4D3). We approximate nuclei as ellipses, and measured the length of the long axis (*L*) and the short axis (*S*) of the nuclei manually using imaging software (ImageJ 1.52a, NIH), and ellipticity (*e*) was calculated as: $e=\sqrt{\frac{L^{2}+S^{2}}{S^{2}}}$. The average ellipticity was statistically-significantly larger for nuclei in the tumor regions (2.6 ± 0.4) compared the periductal (1.7 ± 0.1), lobular (1.6 ± 0.1), and parenchymal (1.6 ± 0.2) regions of normal tissue (*p* < 0.01, *p* < 0.002, and *p* < 0.01, respectively, based on the number of samples). There is not a significant difference in these parameters between nuclei imaged in the periductal, lobular, and parenchymal regions of normal tissue. The nuclear shape and size are easily observed with THG, with essentially no background signal from the extracellular matrix and thus, THG imaging could be used for automated analysis of nuclear size and shape for early cancer detection. The THG intensity was also compared between the different types of tissue, but there was no statistically-significant difference between normal (2,300 ± 1,000) periductal, lobular or parenchymal tissues compared with tumor (3,000 ± 1,500).

## Discussion

Quantitative polarization-resolved SHG values can be used to distinguish between normal and tumor pancreatic tissues. In particular, normal periductal, lobular and parenchymal tissues are significantly different from tumor tissue, as indicated by their *R* values. Additionally, periductal and lobular tissues are significantly different from tumor with respect to the FWHM of *R* occurrence, while parenchymal tissue is significantly different from tumor considering the FWHM of *C* occurrence. Moreover, parenchymal and periductal tissues have significantly different DOLP values from tumor tissue. A limitation of the present study was the relatively small number of patient samples available. However, considering instead the number of regions-of-interest measured, the DOLP values would statistically distinguish lobular tissue from tumor, while the FWHM of the *C* occurrence values would also segregate periductal tissue from tumor. Overall, these results indicate the potential of using polarization SHG parameters for automated cancer diagnosis, for example, by adding PIPO SHG based contrast to histopathology slide scanners, where the SHG parameters for each image pixel in the entire section is determined; regions of concern would then be flagged for pathologist review, based on standardized SHG parameters.

The observed changes in the *R* values for collagenous tissues have been previously attributed to variations in the amino acid content of the triple helices, the arrangement of the triple helices into fibrils and fibers, and the distribution of fibrils and fibers within the laser focal volume (39). We have previously reported similar variations in *R* between normal and tumor tissues in human lung (39), breast (32), and thyroid (42), indicative of increased structural disorder with malignancy. The FWHM of the *C* occurrence parameter is based on the intrinsic chirality of the collagen, but only collagen fibers pointing out of the image plane can have a significant *C* value, so that the observed variations in this parameter likely originate from different angular distributions of the collagen fibers (54).

Several significant variations in the DOLP values were observed across the normal pancreatic tissue types and distinguishes parenchymal from lobular and periductal tissues. Parenchymal tissues had the lowest DOLP values, even lower than in tumor, indicating the most depolarized SHG. Since the tissue samples are thin, scattering of the laser or SHG light or birefringence that would induce elliptical polarization are unlikely to be the dominant effects. Notably, if birefringence was the cause of the DOLP variation, then periductal or lobular tissue would be expected to show a larger effect, since they both have significantly higher collagen content.

Parenchymal tissue is also significantly different than the other tissues when the number of regions measured is considered, rather than the number of patients. In particular, the FWHM of *R* occurrence statistically distinguishes parenchymal from lobular and periductal tissues, while the FWHM of *C* occurrence distinguishes parenchymal from lobular tissues. Interestingly, the *R* parameter does not distinguish between the different normal tissues, indicating that these have similar fiber ultrastructure. On the other hand, the FWHM of *R* occurrence distinguishes between the parenchymal, lobular and periductal tissues, showing some disparity in the collagen. Additionally, the FWHM of *C* occurrence indicates that the ultrastructure of the parenchymal collagen is closer to that of periductal tissue, and that these have differences in collagen fiber angle, α, or chirality as compared to lobular pancreas. The significant DOLP variations, as well as the FWHM of *C* and *R*, are attributed to variations in collagen organization in these tissues. It is known that periductal collagen consists of short concentric segments (61) and parenchymal collagen comprises of short segments, whereas lobular collagen appears to consist of long and straight segments. The measurements here indicate that parenchymal collagen may consist of the shortest segments within the laser focal volume, followed closely by periductal collagen. This distinction of collagen between the periductal, lobular and parenchymal regions is surprising and should be investigated further.

An additional independent cancer marker in pathological tissues treated with H&E dyes is the quantification of nuclei size and shape by performing THG imaging. This information could be used by automated algorithms for differentiating cancer cells. By using a laser excitation of 1030 nm, the THG signals at 343 nm have sufficient transmission through glass for efficient detection, as compared with 800 nm laser excitation, commonly used in non-linear optical imaging, which results in THG signal at 267 nm, where glass absorbs the signals. Therefore, with 1030 nm lasers, SHG and THG can be performed simultaneously without severely affecting the imaging parameters and hence, it is beneficial to combine THG in the analysis, allowing correlations in collagen structure with nuclei size and shape for a more robust cancer diagnosis.

## Conclusions

Human pancreas tissue samples were successfully imaged by polarization-resolved SHG, MPF, and THG microscopies. Using the several polarization SHG parameters (*R*, FWHM of *C* occurrence and DOLP), the normal tissue can be distinguished from tumor tissue. These parameters could be used together for automated cancer detection and as a research tool to understand how the extracellular matrix is formed and evolves with tumor initiation and progression. Further, variations between the collagen structure within normal tissue types were also observed, indicating that collagen structure and ultrastructure vary even within a single organ according to the tissue function. THG microscopy also revealed differences in the nuclear morphology. Combined SHG and THG imaging can be applied for automated cancer detection and can also be used to study the interaction of cancer cells with collagenous structures of the extracellular matrix.

## Ethics Statement

This study was carried out in accordance with the recommendations of the Tri-Council Policy Statement and the University Health Network Research Ethics Board. Additional written consent for this study was not needed to be obtained because the tissues used in this study were collected from a biobank where consent had already been received. The protocol was approved by the University Health Network Research Ethics Board.

## Author Contributions

DT, RC, SA, BW, and VB: project concept and design. DT, RC, and SA: tissue collection and curation. DT, RC, AJ, and SA: formal analysis. BW and VB: funding acquisition. DT, RC, AG, KM, and SK: methodology. DT, RC, and VB: validation. DT and AJ: visualization. DT, RC, and VB: manuscript writing. DT, RC, AJ, AG, KM, SK, SA, BW, and VB: manuscript editing.

### Conflict of Interest Statement

The authors declare that the research was conducted in the absence of any commercial or financial relationships that could be construed as a potential conflict of interest.
